# Upper Thoracic Spine (D2-D3) Fracture With Unilateral Lock Facets Without Associated Neurological Deficits: Case Report & Literature Review

**DOI:** 10.2147/IMCRJ.S412949

**Published:** 2024-01-06

**Authors:** Mohammad Mohsin Arshad, Muhammad Mohsin Khan, Ghanem Al Sulaiti, Ghaya Al Rumaihi

**Affiliations:** 1 Department of Neurosurgery, Neuroscience Institute, Hamad Medical Corporation, Doha, Qatar

**Keywords:** unilateral locked facet, upper thoracic spine, spinal trauma, thoracic dislocation, fracture, spine

## Abstract

**Introduction:**

Upper thoracic spine fractures are rare as compared to other spine segments due to anatomical landmarks. If they occur, they are usually associated with paraplegia or any other neurological dysfunction. We report upper thoracic fracture without neurological dysfunction which is a rare entity along with its radiological imaging, and management plan.

**Case Description:**

Forty-years old male presented after RTA. CT spine showed T2 vertebral body fracture with dislocation/locking of the right T2-T3 facet joint. The patient underwent surgical fixation and was neurologically intact.

**Conclusion:**

Upper thoracic spine fracture is a rare entity due to its anatomical location. And sometimes it is missed as well. Proper imaging should be considered if there is high suspicion and early surgery is warranted to prevent permanent damage.

## Introduction

Unlike the cervical and lumbar spine which allows significant mobility in all axis and thus have a higher predilection to fractures and facet joint injuries than the thoracic spine. This stability of the thoracic spine is due surrounding rib cage and the sagittal orientation of facet joints. As the anatomically thoracic segment has a narrow spinal canal, any injury in this area leads to complete neurological dysfunction in almost 80% of cases.^1^ Reported cases of thoracic spine subluxation with unilateral locked facets without neurological deficits are very rarely found in the literature.

We report a case of upper thoracic spine subluxation with a locked facet joint at the level of D2-D3 without associated neurological deficits in a young patient. The lack of such reported cases makes it difficult to decide its management in such patients. This rare case has been described with specific emphasis on diagnostic radiological features, and management strategies with follow-up planning.

## Case Presentation

**Figure 1:**
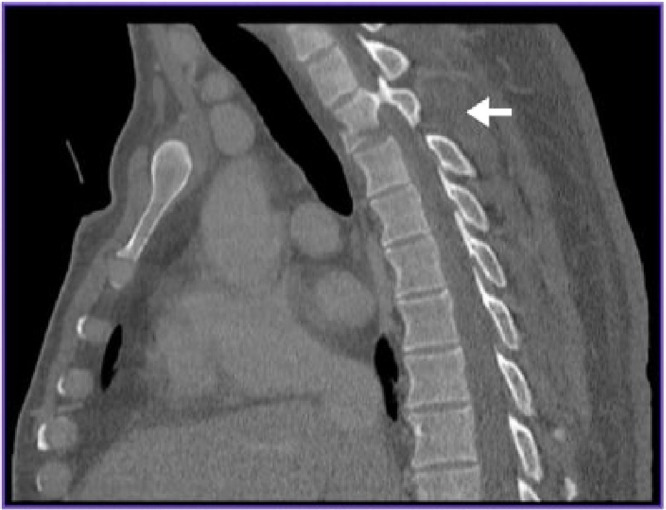
Sagittal computed tomography (CT) image with T2 vertebral body fracture with locking of the right T2-T3 facet joint (Arrow).

**Figure 2:**
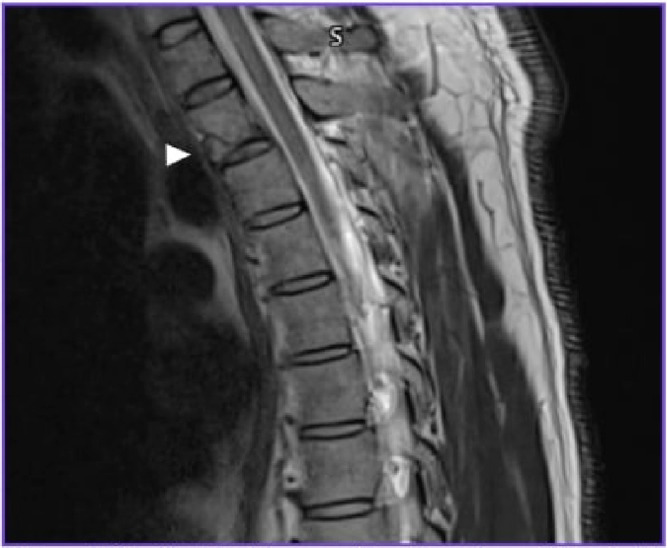
Sagittal MRI image of subluxation of the right T2/3 facet join and teardrop fracture of T2 was also noted (Arrowhead).

A forty-year-old male patient was brought to the hospital as a victim of a motor vehicle rear-end collision. He lost consciousness on impact and was entrapped between the driver seat and the steering wheel. His neurological evaluation revealed no other significant injuries, and according to the ASIA impairment scale, it was grade E. The thoracic computed tomography (CT) showed (Figure 1) a T2 vertebral body fracture with dislocation/locking of the right T2-T3 facet joint. The magnetic resonance imaging (MRI) reported mild anterior displacement of T2 over T3. Subluxation of the right D2/3 facet joint and a teardrop fracture of T2 was also noted. No intramedullary abnormal signal intensity was seen (Figure 2).

The patient underwent surgery for posterior spinal stabilization and pedicle screw fixation from the T1-T3 level with T2 laminae decompression. Intraoperatively, jumped facet was seen between T3 over T2 on the right side with the injured posterior ligament.

**Figure 3:**
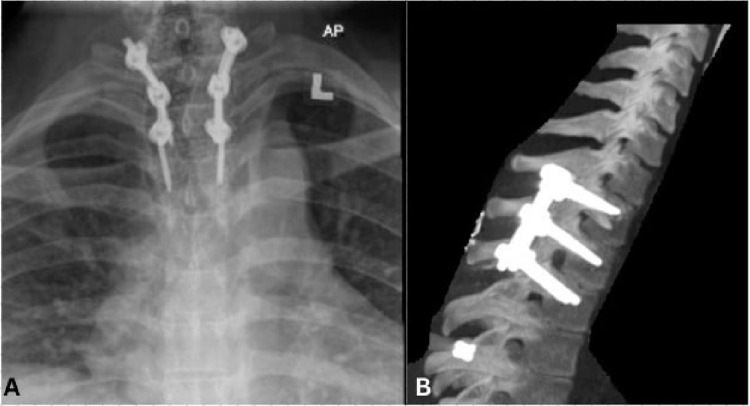
Post-operative AP (**A**) and lateral (**B**) radiograph shows a good alignment of the spinal column following posterior pedicle screw fixation.

The patient was made to walk after being mobilized with the spinal brace on the second postoperative day while still having neurological integrity. After reviewing satisfactory post-operative radiographs (Figure 3), the patient was instructed to wear the spinal brace for three months.

## Discussion

Anatomically, the thoracic spine is considered the strongest and most stable spinal column as compared to the cervical and lumbar spine. The reason for its stability is due to the fact of the presence of the restraining effect of surrounding ribs and costotransverse ligaments, facet joint sagittal orientation, and strong ligamentum flavum with posterior longitudinal ligament complex.^2^ Thus, only trauma with significant force and high energy can disrupt the thoracic spine and its facet joints. Compared to the upper and lower thoracic spine, the mid-thoracic level (T6-9.5mm) has the longest posterior dura-spinal cord distance (T1- 4.7mm and T12- 3.7mm).^3^ Because of high-impact injury and narrow spinal canal, eighty percent of patients with thoracic spine fractures have complete paraplegia.^1^ Interestingly, despite being presented to the hospital with such a severe injury, our patient was neurologically intact. This is likely owing to the pedicle and lamina fracture at D3, which enlarged the spinal canal and prevented spinal cord damage. Roaf et al^4^ mentioned, fracture-dislocation is not created by only hyperflexion and hyperextension alone, but additional rotation forces are required to disrupt all three columns.

Since they require substantially greater axial rotation to rotate around the unaffected facet joint, unilaterally jumped facets in the thoracic spine are much less prevalent than bilateral ones. Unilateral thoracic jumped facets rarely cause cord compression or neurologic symptoms due to the lack of substantial listhesis, but bilateral jumped facets are more likely to cause paraplegia. There is a 25% probability that the unilaterally jumped facet in the cervical spine will sustain neurologic damage.^5^ Table 1^6–10^ summarized all the reported cases of patients with unilateral thoracic jumped facets that were neurological intact except one case, and all were treated by surgical fixation of the thoracic spine except one case of Lucas with D9-10 jumped facet with minimal anterolisthesis.

**Table 1:** Reported Cases of Thoracic Unilateral Jumped Facets

Study	Age	Level	Facet Involved	Mechanism	Neurologic Status	Management
Denis, 1991^7^	22	D3-4	Left	RTA	Intact	Surgical fixation
Denis, 1991^7^	34	D6-7	Right	RTA	Intact	Surgical fixation
Berg, 1991^6^	19	D8-9	Left	RTA	Intact	Surgical fixation
Lucas, 1997^9^	24	D2-3	Right	RTA	Intact	Surgical fixation
Lucas, 1997^9^	19	D8-9	Left	RTA	Intact	Surgical fixation
Lucas, 1997^9^	16	D9-10	Left	RTA	Intact	Bedrest and brace
Gharib, 2002^8^	20	D3-4	Right	RTA	Paraplegic	–
Liu, 2020^10^	45	D2-3	Left	RTA	Intact	Surgical fixation
Our Case	40	D2-3	Right	RTA	Intact	Surgical fixation

**Abbreviation**: RTA, Road traffic accident.

In addition to being uncommon, upper thoracic spine fractures are difficult to diagnose because of the nearby ribs and shoulders that are overlaid on radiographs of this area; many instances have previously been incorrectly identified. Sharafuddin et al^11^ reported that one patient with D2-3 locked facet was misdiagnosed initially on routine workup. It is discovered that plain spine x-rays are inadequate, particularly when the upper dorsal spine is the source of injury. For thoracolumbar spinal injuries, CT is the preferred screening method, although MRI is better for carefully examining the soft tissue anatomy. In our experience, both CT and MRI are helpful for patient management and surgical preparation.

Managing fractures at this zone are difficult due to the transition from a mobile cervical spine to a stationary upper thoracic spine. It is also associated with multiple other injuries, especially of the chest as the high force of impact is involved to cause a fracture in this region.^12^

To prevent future subluxation and consequent cord injury, the primary goal of surgery is to restore spinal stability. Given the fact that it is surrounded by substantial vascular and visceral tissues, the approach in this region is more technically challenging. An anterior approach obviates the need for sternotomy and thoracotomy and a preoperative midsagittal cervicothoracic MRI is required to determine whether the manubrium is an obstacle and to avoid harm to important structures. For anterior spinal fusion of the cervicothoracic junction and upper thoracic spine, preoperative MRI can be a helpful tool.^13^ Posterior instrumentation is also difficult at the cervicothoracic region because of the shoulder and chest wall. Furthermore, the pedicle diameter is too small to put a pedicle screw at this region, and the cervicothoracic curvature transition is very acute at this region. However, recent technical advances have made posterior instrumentation safer and more effective.^14^

Each patient’s particular needs must be taken into consideration when deciding whether to do internal fixation and fusion using anterior, posterior, or both approaches. In our situation, laminectomy and posterior spinal stabilization were performed at D2. The patient began walking the very next day while wearing a spinal thoracic brace after surgery, and his neurological condition remained unaffected.

## Conclusion

Upper thoracic spine subluxation is very rare and in patients with this type of injury, spinal cord is always injured due to a narrow spinal canal dimension, unless there is associated posterior neural arch disruption which widens the spinal canal dimension like in our case. Unilateral thoracic jumped facet should be considered unstable as a large amount of force is required for facet dislocation. CT and MRI should always be considered for early detection of injury in the upper thoracic spine and surgical planning. Early surgery should be considered the first line of treatment in such cases because of spine instability. Cervicothoracic junction instability should be addressed by circumferential fusion, but the surgery is difficult due to unique anatomy and technical restrictions. An in-depth understanding of the benefits and drawbacks of various instrumentation systems can aid in avoiding unforeseen instrumentation constraints and surgical complications.

## Study Settings and Location

Hamad General Hospital – Surgical specialty center, Department of Neurosurgery.

## Approval

Institutional approval was taken for this manuscript.
